# The age of heterozygous *telomerase *mutant **parents influences the adult phenotype of their offspring irrespective of genotype in zebrafish

**DOI:** 10.12688/wellcomeopenres.12530.2

**Published:** 2018-02-22

**Authors:** Catherine M. Scahill, Zsofia Digby, Ian M. Sealy, Richard J. White, Neha Wali, John E. Collins, Derek L. Stemple, Elisabeth M. Busch-Nentwich

**Affiliations:** 1 Wellcome Trust Sanger Institute, Wellcome Genome Campus, Hinxton, UK; 2 Department of Veterinary Medicine, University of Cambridge, Cambridge, UK; 3 Department of Medicine, University of Cambridge, Cambridge, UK

**Keywords:** telomerase, telomere length, zebrafish, age, sex, Tert

## Abstract

Background: Mutations in proteins involved in telomere maintenance lead to a range of human diseases, including dyskeratosis congenita, idiopathic pulmonary fibrosis and cancer. Telomerase functions to add telomeric repeats back onto the ends of chromosomes, however non-canonical roles of components of telomerase have recently been suggested.

Methods: Here we use a zebrafish telomerase mutant which harbours a nonsense mutation in *tert* to investigate the adult phenotypes of fish derived from heterozygous parents of different ages. Furthermore we use whole genome sequencing data to estimate average telomere lengths.

Results: We show that homozygous offspring from older heterozygotes exhibit signs of body wasting at a younger age than those of younger parents, and that offspring of older heterozygous parents weigh less irrespective of genotype. We also demonstrate that *tert* homozygous mutant fish have a male sex bias, and that clutches from older parents also have a male sex bias in the heterozygous and wild-type populations. Telomere length analysis reveals that the telomeres of younger heterozygous parents are shorter than those of older heterozygous parents.

Conclusions: These data indicate that the phenotypes observed in offspring from older parents cannot be explained by telomere length. Instead we propose that Tert functions outside of telomere length maintenance in an age-dependent manner to influence the adult phenotypes of the next generation.

## Introduction

Telomeres are composed of non-coding repetitive sequences at the termini of each chromosome, protected by a complex of proteins called the shelterin complex. With each cell division the telomeres shorten due to the inability of DNA polymerases to replicate the ends of linear DNA, a phenomenon known as the ‘end replication problem’ (reviewed in 1). To circumvent this problem certain cell types express the ribonucleoprotein Telomerase, which adds telomeric repeats back onto the ends of chromosomes (reviewed in 2). When telomeres lose their protective cap, the linear DNA ends are recognised by DNA damage response proteins ^3^ resulting in telomere fusions ^4– 6^ and ultimately leading to replicative senescence and cell death ^7– 9^.

Telomerase is composed of an RNA molecule ( *hTR* in humans, *terc* in zebrafish) containing a complementary sequence to the telomeric repeat hexamer which serves as a template for DNA synthesis ^10^ and a reverse transcriptase enzyme (TERT in humans, Tert in zebrafish) which harbours an RNA binding domain and a reverse transcriptase domain ^11– 13^.

Mutations in components of telomerase or other telomere-associated proteins lead to a class of human diseases collectively known as telomeropathies (reviewed in 14, 15). These include dyskeratosis congenita (DC), idiopathic pulmonary fibrosis (IPF) ^16, 17^ and cancer ^18^. Telomerase is not expressed in most somatic cells but its expression is activated in germline tissue and tumour cells ^10, 19^ enabling these cell populations to divide potentially indefinitely, and thus activation of telomerase is a hallmark of many cancers ^18^. DC results from germline mutation in a number of telomere-associated genes including *DKC* ^20– 22^, *TINF2* ^23, 24^, *TERC* ^25^ and *TERT* ^26^, and its clinical features combine IPF, cancer predisposition, skin abnormalities and bone marrow failure. Interestingly, patients with autosomal dominant DC show disease anticipation, due to haploinsufficiency of either *TERT* or *TERC*, whereby the age of onset and severity of symptoms worsens in successive generations ^27, 28^.

Mouse models were traditionally used to study telomere dysfunction, however laboratory mouse strains have very long telomeres ^29, 30^ and consequently *mTR* and *mTERT* homozygous mutants can be incrossed for several generations before phenotypes become apparent ^6, 31^, although in the case of *mTERT* null mutants, genetic background contributes significantly to the generation in which phenotypes arise ^32^. More recently zebrafish models have been used to interrogate the function of telomerase in development and disease. Fish homozygous for the null mutation *tert ^hu3430^* have shorter telomeres and show premature lethality and tissue degeneration. In addition, a p53-mediated reduction in cell proliferation and increased apoptosis are observed in these mutants ^33, 34^.

Here we have characterised a new nonsense mutation in the zebrafish *tert* gene, *tert ^sa6541^*, and show that the age of heterozygous parents influenced the adult phenotypes of their offspring. Offspring of all genotypes from older parents weighed less and showed a male sex bias. We used whole genome sequencing to estimate average telomere lengths and surprisingly found that the phenotypes observed were independent of telomere length. This highlights the need for further investigation into the additional roles of telomerase aside from maintaining telomere length.

## Methods

### Husbandry

This study was conducted in line with the ARRIVE guidelines ^35^. Zebrafish were maintained in accordance with UK Home Office regulations, UK Animals (Scientific Procedures) Act 1986, under project licence 70/7606, which was reviewed by the Wellcome Trust Sanger Institute Ethical Review Committee. The mutant alleles *tert ^sa6541^* and *tert ^sa25076^* were obtained from the Zebrafish Mutation Project ^36^ and recovered from frozen sperm samples at the Wellcome Trust Sanger Institute. Embryos were produced through natural matings and maintained in an incubator at 28.5°C up to 5 days post fertilisation (d.p.f.). All efforts were made to ameliorate any suffering: fish underwent careful health checks twice a day and any fish that showed signs of distress, such as lethargy, failure to feed or abnormal swimming behaviour, were culled following the standard Schedule 1 method of anaesthetic overdose with subsequent destruction of the brain. Only morphologically normal larvae with inflated swim bladders at 5 d.p.f. entered the nursery to be raised to adulthood for this study.

### Genotyping

DNA from embryos or fin biopsies was extracted and genotyped for *tert ^sa6541^* or *tert ^sa25076^* using KASP genotyping as previously described ^37^.

### Sperm extraction

Males were anaesthetised and sperm extracted by gentle abdominal massage as described previously ^37^.

### Weight measurements

Anaesthetised males were patted dry and weighed in a dish of system water on electronic scales.

### Telomere length analysis

DNA was extracted from caudal fin clips and used to make Illumina libraries which were sequenced on four lanes of HiSeq X in 151 bp paired-end mode. Sequence data were deposited in ENA under accession ERP016250. FASTQ files were analysed using Computel ^38^ (v0.3, options: -proc 4 -nchr 25 -lgenome 1371719383 -pattern TTAGGG). FASTQ files were also aligned to the Zv9 reference genome using BWA (v0.7.15, options: mem -t 14 -p -Y -K 100000000) and analysed using TelomereHunter ^39^ (v1.0.4, options: -d –r TTAGGG -nf).

## Results

### *tert ^sa6541/sa6541^* fish age prematurely

**Figure 1.**
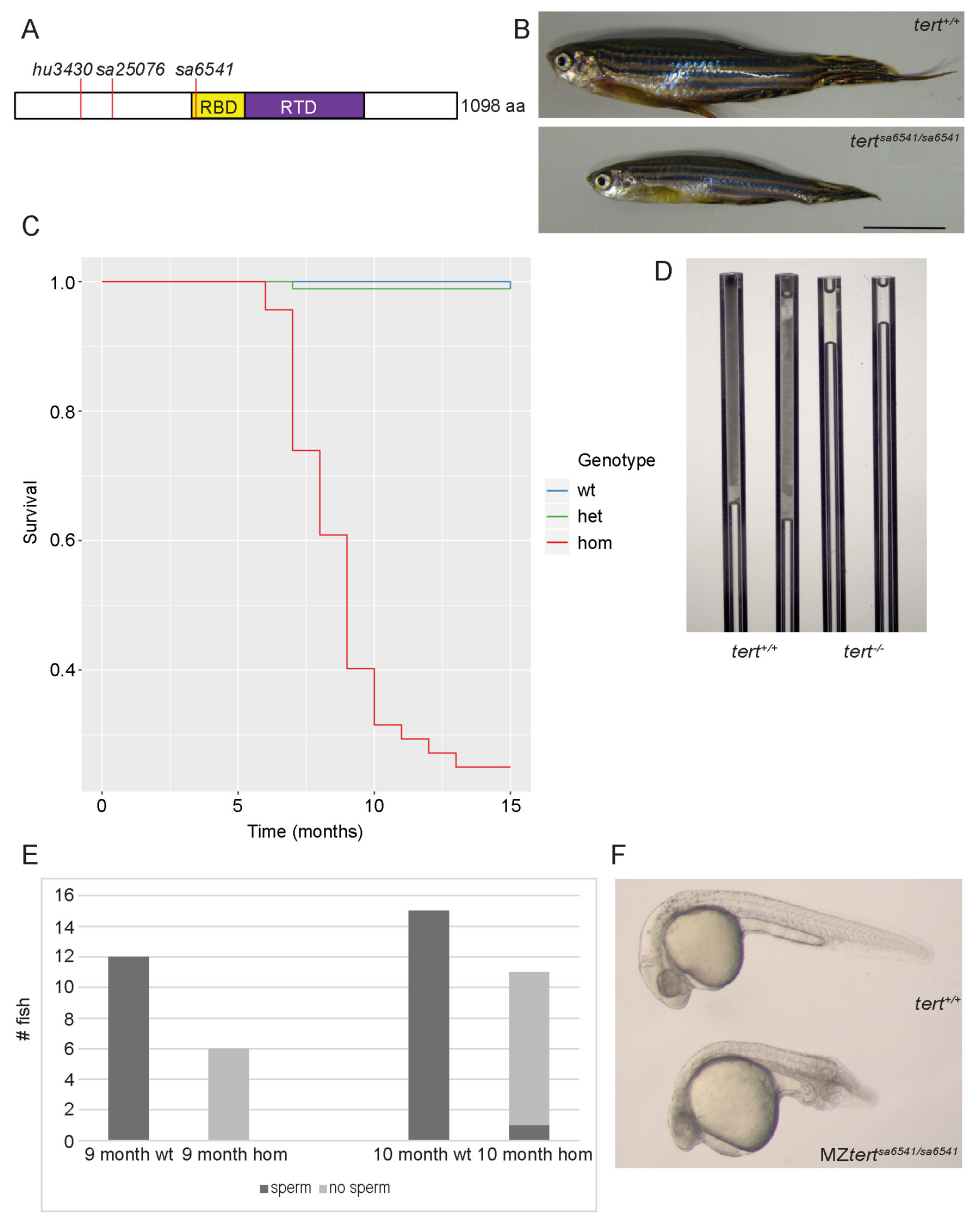
*tert ^sa6541/sa6541^* fish age prematurely. ( **A**) Protein domain structure of zebrafish Tert. Depicted in yellow is the telomerase ribonucleoprotein complex RNA-binding domain (RBD), and in purple the reverse transcriptase domain (RTD). The position of three known alleles are shown. ( **B**) *tert ^sa6541/sa6541^* fish age prematurely. Homozygous fish display a wasting phenotype. Scale bar: 10mm. ( **C**) *tert ^sa6541/sa6541^* fish die prematurely compared to their siblings (n=92 homozygotes, n=92 heterozygotes, n=92 wild-types). ( **D**) Photograph of capillaries containing sperm from wild-type sibling males (left) and clear fluid containing no sperm from *tert ^sa6541/sa6541^* males (right). ( **E**) Homozygous *tert ^sa6541^* fish become prematurely infertile. All wild-type males but only 5.9% of homozygous males tested, aged 9 or 10 months, produced sperm. ( **F**) Maternal-zygotic mutant embryos derived from *tert ^sa6541/sa6541^* intercrosses display a range of phenotypes by 24 h.p.f. including a reduction in head tissue and a shorter tail.

Fish homozygous for the point mutation *tert ^hu3430^* age prematurely, evidenced by spinal curvature, tissue degeneration and premature infertility and lethality ^33, 34^. We have characterised a new *tert* allele *, tert ^sa6541^*, produced by the Zebrafish Mutation Project ^36^, which carries a point mutation resulting in a premature stop codon and a predicted protein lacking the reverse transcriptase domain and most of the RNA-binding domain ( Figure 1A). We compared the phenotype of *tert ^sa6541/sa6541^* fish to the published phenotype for *tert ^hu3430/hu3430^* to assess whether *tert ^sa6541^* recapitulates the published zebrafish *tert* knockout phenotype ^33, 34^. Fish homozygous for *tert ^sa6541^* show visible signs of ageing earlier than their siblings, including a wasting phenotype ( Figure 1B), and homozygotes die prematurely ( Figure 1C). As previously shown for *tert ^hu3430^*, *tert ^sa6541^* homozygous males become prematurely infertile; by 9–10 months of age, most (16/17) homozygous males failed to produce any sperm by gentle abdominal massage, whereas all (27/27) wild-type siblings produced sperm ( Figure 1D and E). Furthermore maternal zygotic *tert ^sa6541/sa6541^* fish displayed a variety of embryonic phenotypes ( Figure 1F) in accordance with the previously published phenotypes. Intercrosses from heterozygous fish (965 embryos from 3 separate clutches) showed no significant difference (Welch two sample t-test, p-val=0.49) in the number of phenotypic embryos compared to wild-type sibling intercrosses (614 embryos from 4 separate clutches).

To further confirm that the phenotypes observed in *tert ^sa6541/sa6541^* fish were due to the mutation in *tert*, we generated compound heterozygous fish using another uncharacterised *tert* allele. *tert ^sa25076^* carries a point mutation resulting in a premature stop codon and a predicted protein lacking both the reverse transcriptase and RNA binding domains ( Figure 1A). Embryos from compound heterozygous intercrosses displayed a range of phenotypes similar to those observed in *tert ^sa6541/sa6541^* intercrosses ( Supplementary Figure S1A) and by 5 days post fertilisation (d.p.f.) compound heterozygous intercrosses had fewer non-phenotypic embryos compared to wild-type siblings ( Supplementary Figure S1B).

### The age of heterozygous *tert ^sa6541^* parents influences the adult phenotype of their offspring

**Figure 2.**
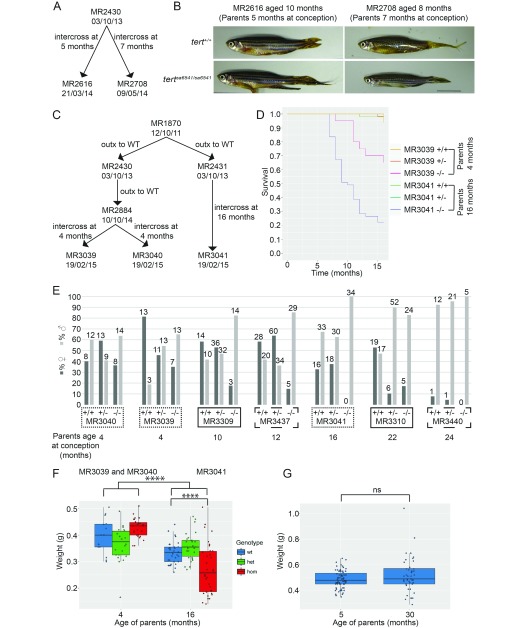
The age of heterozygous *tert ^sa6541/+^* parents affects the phenotype of their offspring. ( **A**) Family tree depicting relationship between lines shown in ( **B**). Lines numbers are indicated according to our fish stock database with date of birth underneath. ( **B**) Homozygous fish from older heterozygous parents show signs of body wasting at a younger age than those from younger heterozygous parents. 8 month old fish from 7 month old heterozygous parents appear older than 10 month old fish from 5 month old heterozygous parents. Scale bar: 10mm. ( **C**) Family tree depicting relationship of lines shown in ( **D**) and ( **F**). ( **D**) Homozygous fish from 16 month old heterozygous parents have reduced survival compared to homozygotes from 4 month old heterozygous parents which were raised at the same time. ( **E**) Sex ratios of intercrosses from *tert ^sa6541/^* ^+^ parents of different ages. Patterned boxes around line numbers indicate which lines were raised simultaneously in the nursery. Absolute numbers are indicated above each bar. ( **F**) Box plot with scatter of weights of male fish from 4 or 16 month old heterozygous parents showing that fish from older parents weigh less than those from younger parents. ( **G**) Box plot with scatter showing no significant difference between the weights of wild-type fish from wild-type parents aged 5 or 30 months.

We observed when intercrossing heterozygous fish over several months ( Figure 2A), that lines produced from older parents showed signs, at a younger age, of body wasting reminiscent of fish aged 18 months or older ( Figure 2B). Homozygous fish from older parents were less plump (note sunken abdomen in 8 month old homozygote) and paler than older homozygotes derived from earlier matings. This led us to investigate the effect of *tert ^sa6541/+^* parental age on the phenotype of their offspring.

We raised three *tert ^sa6541/+^* lines; MR2430 and MR2431, which were the same age, and MR2884, which was 12 months younger and obtained from an outcross of MR2430 (see Supplementary Figure S2 for full family tree). We intercrossed MR2431 and MR2884 at 16 and 4 months of age respectively and raised their offspring alongside each other in the nursery to minimise the effect of environmental influences on survival. This revealed that homozygous fish from older parents had reduced survival compared to homozygous fish from younger parents. By contrast, survival of heterozygous and wild-type siblings was unaffected by parental age ( Figure 2D). However comparing survival rates across multiple lines raised at different time points showed that this was not a consistent phenotype ( Supplementary Figure S3). This may reflect the many different factors that contribute to survival.

Furthermore we observed an effect of heterozygous parental age on the sex ratios of their offspring. Irrespective of the age of the parents, there was a strong male sex bias among *tert ^sa6541/sa6541^* fish ( Figure 2E). However as the age of the parents increased, this male sex bias became more pronounced. While 23/93 (24.7%) homozygotes were female in fish conceived at 12 months or younger, only 5/68 (7.4%) homozygous fish conceived at 16 months or older were female. Table 1 provides a list of the sex ratios compared to the expected ratio of 0.5 with significant deviations marked with an asterisk. The mechanisms of sex determination in zebrafish are poorly characterised and are influenced by environmental factors ^40– 42^. Nevertheless, the sex bias in *tert ^sa6541^* homozygous fish is specific to mutation in *tert* since lines produced from younger parents (12 months and below) have heterozygous and wild-type sibling populations that do not differ significantly from a 0.5 sex ratio with 120/208 (57.7%) and 63/108 (58.3%) females, respectively. This indicates that the male skew in homozygous fish is not a result of environmental conditions during the raising of the line. However, with advanced age of the heterozygous parents, a male sex bias also became apparent in heterozygous and wild-type offspring populations ( Figure 2E). In heterozygous and wild-type fish from parents aged 16 months and older the female proportions dropped to 25/128 (19.5%) and 36/98 (36.7%) respectively. These fish were raised alongside lines from younger parents to eliminate environmental influence (indicated as different rectangles in Figure 2E). It is conceivable that heterozygous females have an oogenesis defect with downstream consequences for development. However, we did not find a statistically significant difference in clutch size (Welch two sample t-test, p-val=0.53) or fertilisation rates (Welch two sample t-test, p-val=0.14) between wild-type and heterozygous intercrosses ( Supplementary Figure S4A). In addition, we investigated whether there was a general influence of parental age on sex ratios across lines raised in our facility. There was no measurable trend to either sex correlating with the age of either parent ( Supplementary Figure S4B). In conclusion these data indicate that the age of heterozygous parents affects sex determination in their offspring.

**Table 1.** Sex ratios of fish from old or young heterozygous parents. The sex ratio of fish with each genotype (GT) from young parents aged 12 months or younger, and old parents aged 16 months or older was compared to 0.5 using a Chi-squared test. The p-values were adjusted for multiple testing using Bonferroni correction. (*) next to the adjusted p-value indicates significance at p<0.05.

Parental age at conception (months)	GT	# fish	# females	% female	p-val	Adj. p-val
12 or younger	wt	108	63	58.3	0.083	0.5
	het	208	120	57.7	0.027	0.159
	hom	93	23	24.7	1.10E-06	6.57E-06 *
16 or older	wt	98	36	36.7	0.009	0.052
	het	128	25	19.5	5.41E-12	3.25E-11 *
	hom	68	5	7.4	2.01E-12	1.21E-11 *

We observed that *tert* homozygous fish from older heterozygous parents appeared smaller than those from younger parents when raised alongside each other. Given the sex bias in clutches produced from older parents we quantified the weights of 8 month old adult males from MR3039 and MR3040, which had parents aged 4 months at the time of conception, and 8 month old MR3041 males, which had 16 month old parents at the time of conception ( Figure 2C). This revealed that all fish from 16 month old parents, irrespective of genotype, were significantly smaller than those from 4 month old parents (linear model formula: weight ~ genotype * parental age; p-value: <10 ^-16^) ( Figure 2F). Additionally, *tert ^sa6541/sa6541^* fish produced from intercrosses of older parents were also significantly smaller than their wild-type siblings (linear model formula: weight ~ genotype * parental age; p-value: 0.0001) ( Figure 2F). This indicates that older *tert ^sa6541/+^* parents produce smaller offspring irrespective of genotype, and that the effect of being homozygous is only significant when these fish have older parents. To assess whether smaller offspring was a general phenotype of older fish, we intercrossed wild-type fish aged 5 and 30 months, but found no significant difference between the weights of their male offspring ( Figure 2G) suggesting that the size difference observed between the two *tert ^sa6541^* lines is a consequence of the interaction of parental *tert* heterozygosity with age.

### Phenotypes observed in fish from older heterozygous parents are independent of telomere length

**Figure 3.**
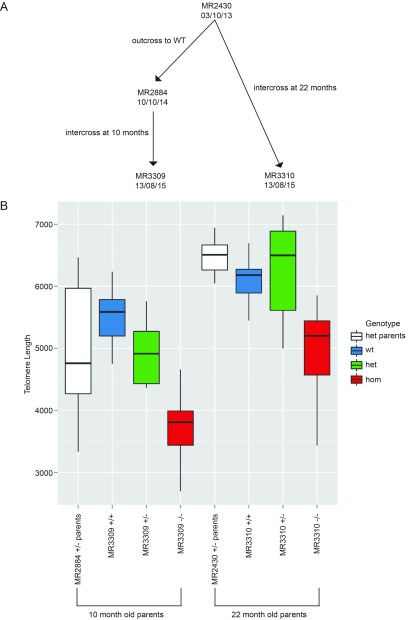
Telomere lengths of families with old or young parents. ( **A**) Family tree depicting the relationship of the lines used in the telomere length analysis. ( **B**) Box plot of average telomere lengths, estimated using Computel, of fish from two families, one with heterozygous parents aged 22 months and one with heterozygous parents aged 10 months.

To assess whether the reduced body weight and more pronounced male sex bias observed in adult fish from older heterozygous parents were a result of shorter telomere lengths, we used whole genome sequencing to estimate telomere lengths; a method which has been shown to produce comparable results to other methods of measuring telomere lengths such as Southern blot ^43^ and qPCR ^38^. We extracted DNA from caudal fin clips, performed whole genome sequencing (mean coverage 6x) and determined average telomere length per sample using Computel ^38^. We fin clipped 8 fish from each of: an old *tert ^sa6541^* heterozygous stock (MR2430) which was 30 months old at the time of clipping; adult offspring of each genotype from MR3310, which were 7 months old at the time of clipping and were the progeny of an intercross of MR2430 at 22 months; a younger *tert ^sa6541^* heterozygous stock (MR2884) which was 17 months old at the time of clipping; and adult offspring of each genotype from MR3309, which were 7 months old when clipped and were the result of an intercross of MR2884 at 10 months ( Figure 3A). Both intercrosses (MR3309 and MR3310) were raised at the same time to minimise the effect of environment on telomere length.

Telomere length data were analysed using one-way ANOVA, followed by Tukey’s test. For both parent-offspring families, the homozygous fish had significantly shorter telomeres than their parents (MR3309 p=0.01, MR3310 p=0.001) and both their wild-type (MR3309 p=0.0001, MR3310 p=0.03) and heterozygous (MR3309 p=0.02, MR3310 p=0.006) siblings ( Figure 3B). Similarly *tert ^hu3430/hu3430^* fish have been shown to have shorter telomeres than their wild-type siblings ^33^.

Surprisingly, the older parental heterozygous stock had longer telomeres than the younger parental heterozygous stock (p=0.002) ( Figure 3B). This is contrary to our expectations since MR2430 was 30 months old at the time of fin clipping whereas MR2884 was only 17 months old. Furthermore, within each parent-offspring family the average telomere length of both the wild-type and the heterozygous offspring did not differ significantly from that of their parents. This indicates both that the heterozygous and wild-type offspring were able to maintain their inherited telomere length and that the phenotypes observed in the heterozygous and wild-type populations from older heterozygous parents are not the consequence of shorter telomeres, considering either the absolute telomere length or the telomere length relative to their parents. The same pattern of telomere lengths was also found when using TelomereHunter ^39^, a tool that determines telomere lengths from sequencing data using a different method to Computel ( Supplementary Figure S5A).

The offspring lines MR3309 and MR3310 used in this telomere length analysis were also used in the sex ratio analysis ( Figure 2E). This enabled us to show specifically that the sex ratios are independent of telomere length. Despite having shorter average telomere lengths, MR3309 wild-type and heterozygous fish did not have a male sex bias. By contrast even though both had longer telomeres, MR3310 heterozygous fish had a strong male sex bias, whereas wild-type fish did not ( Figure 2E).

## Discussion

We have characterised a new mutation in zebrafish *tert, tert ^sa6541^*, and shown that it recapitulates the phenotype of the previously published *tert* knockout *tert ^hu3430^*. We focussed our analysis on the adult phenotypes resulting from *tert* haploinsufficiency in heterozygous parents, and showed that the age of heterozygous parents affected the phenotype of their adult offspring. Importantly, we have demonstrated that these adult phenotypes are not simply a consequence of shorter telomeres in offspring from older parents as one might expect, but instead our data point to a role for Tert independent of telomere lengthening.

There have been a number of studies showing that TERT functions outside of telomere maintenance. TERT is involved in Wnt signalling ^44, 45^, regulation of transcription ^45^ and epithelial cell proliferation ^45, 46^ and haematopoiesis ^47^. Confirmation that TERT acts independently of telomere lengthening in these situations came from studying TERT mutants lacking reverse transcriptase function ^45^ or the ability of a *tert* construct lacking the *terc* binding domain to rescue blood cell numbers in Tert-deficient zebrafish ^47^, or the effects of TERT in a TERC ^-/-^ background ^46^. However the mechanism of TERT function, other than in telomere maintenance, remains poorly understood and which aspects of a TERT-deficient phenotype are due to defects in telomere maintenance and which to non-canonical roles of TERT is an area that requires further investigation.

When two lines from parents of different ages were raised simultaneously we observed reduced survival in homozygous offspring from older *tert ^sa6541^* heterozygous parents but no difference in the survival of wild-type or heterozygous siblings between the two lines. This suggests that *tert* genotype interacting with parental age underlies the reduced survival. However this pattern of reduced survival with increasing parental age cannot be robustly observed when comparing survival across many of the lines raised. This may be due to changes in environmental factors when lines were raised at different times.

We demonstrated that offspring of older *tert ^sa6541^* heterozygous parents weighed less than those from younger parents, and that this weight difference was more pronounced when specifically comparing homozygous offspring to wild-type siblings. This could reflect a slower rate of proliferation or increased apoptosis in fish from older parents. Mouse embryos homozygous mutant for *mTERT* and zebrafish embryos resulting from homozygous *tert ^hu3430^* intercrosses are smaller than their wild-type counterparts ^34, 48^ indicating that lack of TERT can influence size, and increased p53-dependent apoptosis has been shown in both zebrafish *tert* knockouts ^33, 34^ and mouse *mTR* mutants ^49^. However a parental age-dependent effect on body weight has not previously been demonstrated to result from haploinsufficiency in the parental generation. Thus a combination of reduced proliferation and increased apoptosis may explain the reduced body weight of fish from older *tert ^sa6541^* heterozygous parents. It is surprising though that the effects of *tert* haploinsufficiency can affect even wild-type offspring in the next generation which are able to express zygotic Tert.

The strong male sex bias in *tert ^sa6541/sa6541^* fish suggests they have accumulated DNA damage. Mutations in genes involved in DNA damage repair such as *brca2* result in all fish developing as male ^50^. This is due to the developing oocytes dying, presumably as a result of an accumulation of DNA damage resulting from recombination during meiosis, and, without the presence of oocytes, juveniles develop as phenotypically male ^50, 51^. The shorter telomeres of homozygous fish, as revealed by Computel and TelomereHunter analysis of whole genome sequencing data, cannot by themselves explain the accumulation of DNA damage since heterozygous offspring from young parents had a similar average telomere length to homozygous offspring from older parents, but failed to show a male sex bias.

We have used an average telomere length estimate to compare telomere lengths of fin biopsies, but this does not give us the resolution to be able to assess the lengths of individual telomeres. Whilst our data suggest that the phenotypes we observed are independent of telomere length, we cannot rule out the possibility that fish from older heterozygous parents contain one or two chromosomes with critically short telomeres that elicit genomic instability. It has been proposed that the shortest telomere length, not the average, is the most relevant measure for telomere dysfunction ^52^, however average telomere length remains a common measure and has been shown to correlate with health markers and survival in both humans ^53– 59^ and animals ^60, 61^. Furthermore, we only measured telomere lengths of caudal fin biopsies and it is possible that other tissues will behave differently.

The average telomere lengths of older parental heterozygous *tert ^sa6541^* fish was longer than for younger parental heterozygotes. This was in contrast to what we expected, as the general consensus is that telomeres shorten with age ^62– 65^, and there was a 12 month age difference between the two lines. This age difference resulted from an additional outcross to wild type for the younger line. Successive intercrosses of *mTERT* heterozygous mice have progressively shorter telomeres due to *TERT* haploinsufficiency ^48^, but this is unlikely to explain the shorter telomeres in younger *tert ^sa6541^* heterozygous fish. Firstly, we introduced a wild-type copy of each chromosome from our wild-type stock rather than intercrossing carriers for the additional generation, and, secondly, our telomere length measurements showed that wild-type and heterozygous offspring maintained their inherited telomere length, indicating that a shortening of telomeres is not inevitable with each generation of *tert* heterozygous fish. Indeed it has been shown in humans that paternal age at birth is positively correlated with offspring telomere length ^66^. Instead the difference in telomere length suggests that the genetic background of each line plays a significant role in determining telomere length, as has been shown for mice ^32^. More specifically, it is possible that from the distribution of mean telomere lengths in the grandparents, individuals with longer telomeres were crossed to produce MR3310 and an individual with by chance shorter telomeres was crossed to produce MR2884. This notion is supported by the finding that telomere length in the wild-type and heterozygous offspring lies in the range of their respective parents, but is different between the families despite being of the same age.

Haploinsufficiency of either *TERT* or *TERC* causes dyskeratosis congenita (DC) in human patients. Our zebrafish loss of function model recapitulates haploinsufficiency of *tert*, but this haploinsufficiency is not evident in the heterozygous parents themselves, and, instead, heterozygosity of *tert* interacts with parental age to produce phenotypes in the next generation. This suggests that *tert* haploinsufficiency manifests in gametogenesis and progressively worsens as parents age. It is tempting to speculate that offspring from older parents inherit shorter telomeres, leading to phenotypes in offspring from older parents, but our telomere length analysis indicates that this is not the case. How heterozygosity of *tert* in the parental generation can affect the adult phenotype of their offspring in a parental age-dependent manner remains to be elucidated. A detailed analysis of the structure and stability of chromosomes in general, not just focussing on the telomeres, in gametes from parents of different ages will be needed to address this question.

Human diseases resulting from loss of TERT, such as DC, comprise several different phenotypes. In the case of DC, patients suffer from idiopathic pulmonary fibrosis, increased cancer susceptibility, skin pigmentation defects, nail dystrophy and bone marrow failure. Given the emerging evidence for telomere-independent roles of TERT, including the data presented in this study ^44– 47^, dissecting the mechanisms of pathogenesis of different components of such diseases may reveal new areas that could be the focus of disease interventions.

## Data availability

Whole exome sequencing data are available from ENA under Study Accession Number: ERP016250. All other raw data are available via Figshare at https://doi.org/10.6084/m9.figshare.c.3866671 ^67^

## Supplementary material

**Supplementary Figure S1. *tert ^sa6541/sa25076^* compound heterozygous fish display the same phenotype as *tert ^sa6541/sa6541^* homozygous fish.**

(A) Embryos produced from intercrosses of compound heterozygous fish display a range of phenotypes similar to those resulting from *tert ^sa6541/sa6541^* homozygous incrosses. (B) Compound heterozygous intercrosses produce clutches with fewer non-phenotypic embryos at 5 d.p.f. than wild-type sibling intercrosses.

**Supplementary Figure S2. Family tree of *tert ^sa6541^* lines used in this study.**

The relationship between all the *tert ^sa6541^* lines is depicted as a family tree. Line numbers are given according to our fish database with date of birth below.

**Supplementary Figure S3. Survival graphs of progeny from intercrosses of different aged *tert ^sa6541^* heterozygous parents.**

Each graph shows the survival of homozygous, heterozygous and wild-type offspring from individual intercrosses. Parental age is indicated for each intercross.

**Supplementary Figure S4. Clutch characteristics of tert ^sa6541/+^ intercrosses and scatterplot of sex ratios in relation to parental age of 74 different lines.**

**Supplementary Figure S5. Telomere lengths of fish from Figure 3A estimated using TelomereHunter.**
