# Supplementary figures and images for: The age of heterozygous telomerase mutant parents influences the adult phenotype of their offspring irrespective of genotype in zebrafish
Source: Wellcome Open Res. 2018 Feb 22;2:77. Originally published 2017 Sep 4. [Version 2] doi: 10.12688/wellcomeopenres.12530.2 (PMC5840683; doi:10.12688/wellcomeopenres.12530.2)

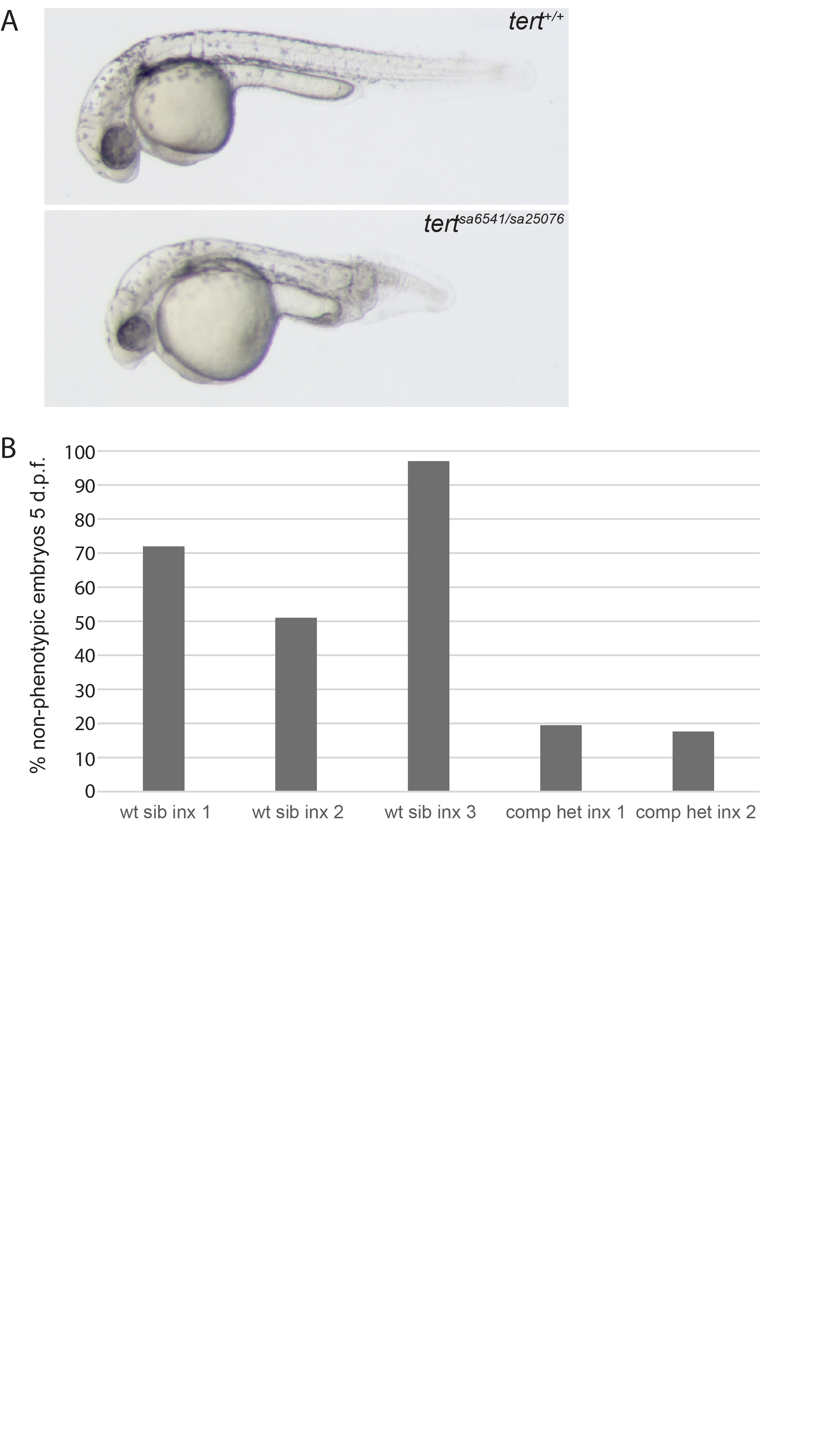

Supplement: Supplementary file 1 [file wellcomeopenres-2-15254-s0000.tgz › e8fe73fd-4151-4e58-bb81-c07bfd062df4.tif]

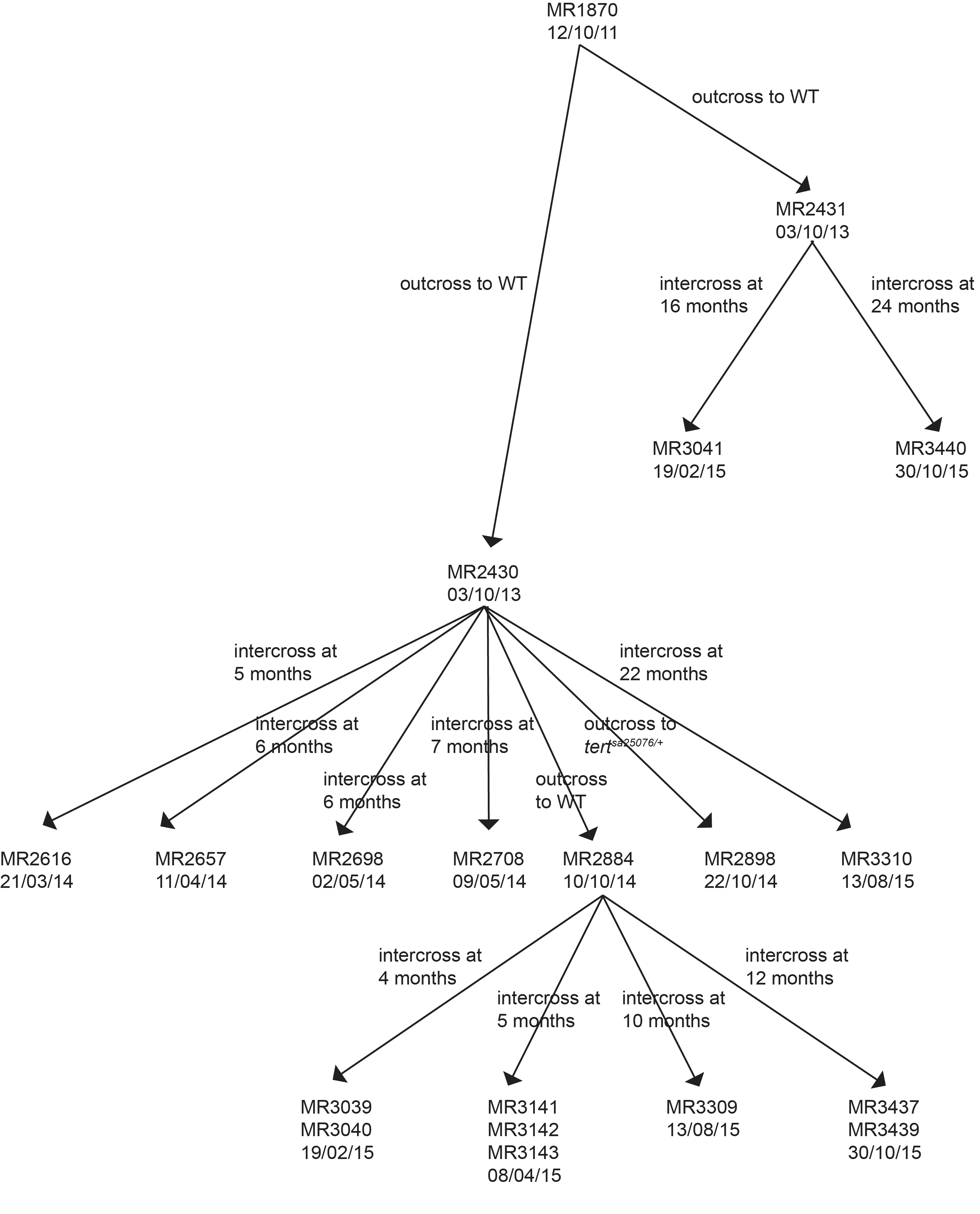

Supplement: Supplementary file 2 [file wellcomeopenres-2-15254-s0001.tgz › 9fecbb95-c6f0-4948-ab4a-61ec353790a9.tif]

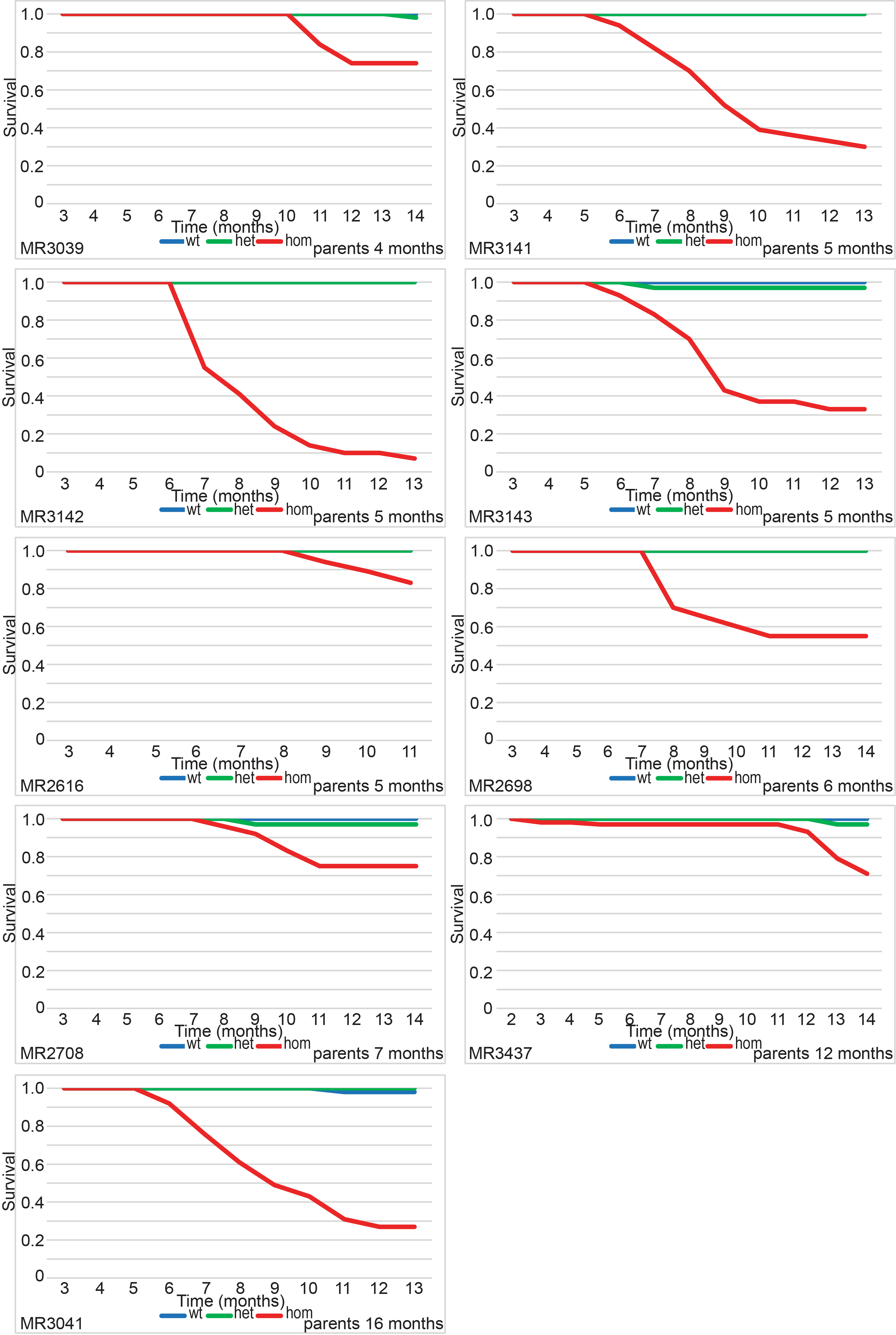

Supplement: Supplementary file 3 [file wellcomeopenres-2-15254-s0002.tgz › 0d991f54-b06c-45a7-8797-32272de61ab4.tif]

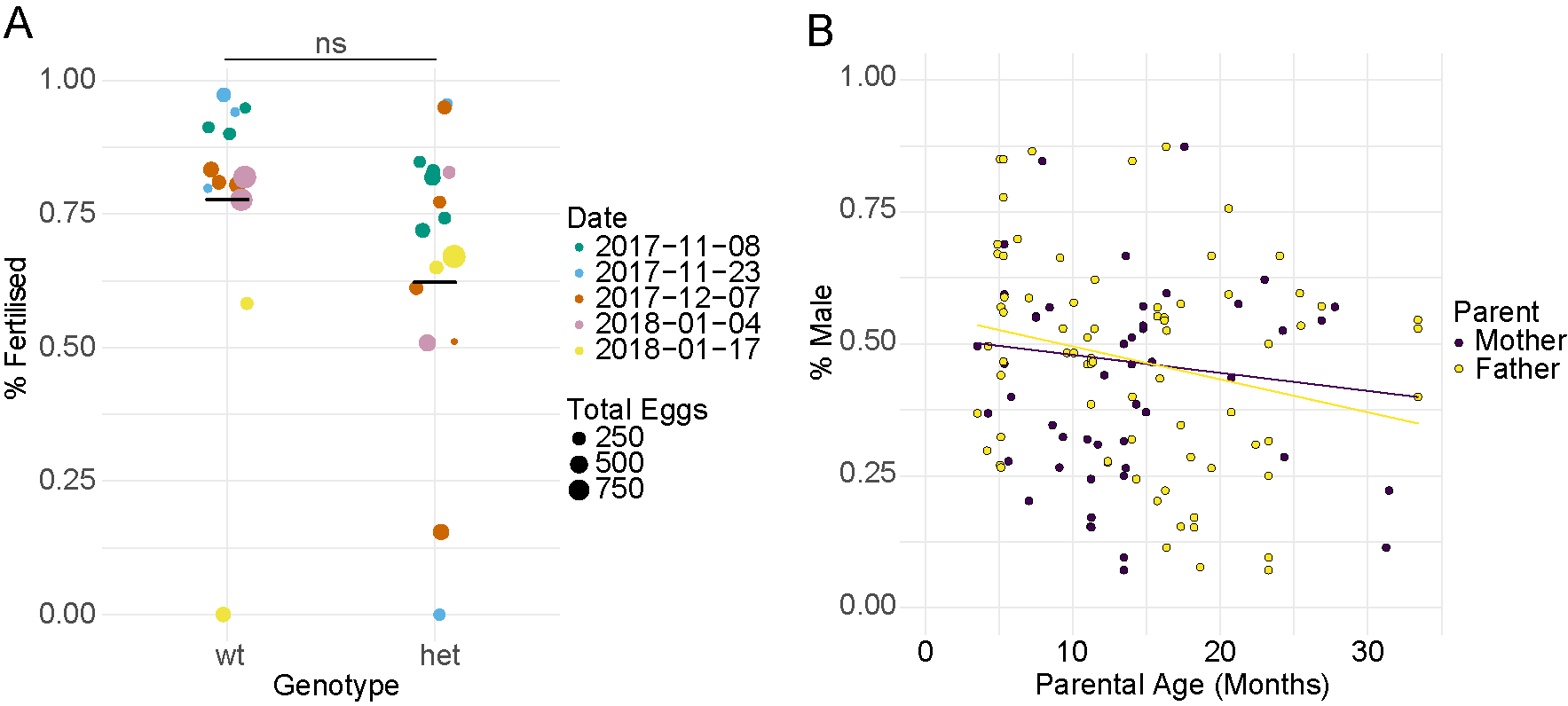

Supplement: Supplementary file 4 [file wellcomeopenres-2-15254-s0003.tgz › 39c9ccd0-d8fa-409f-b128-be2a0bc34b34.tif]

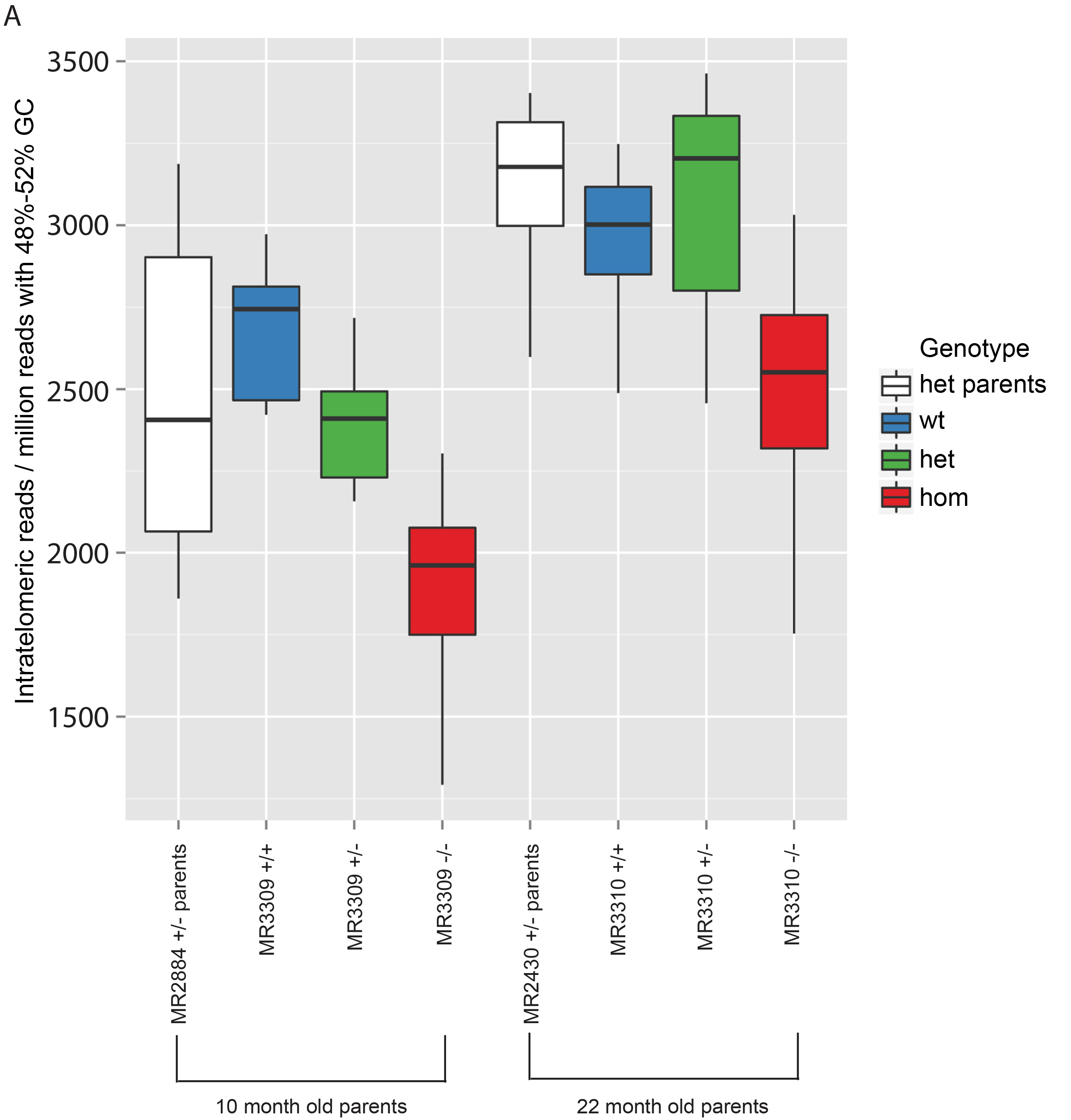

Supplement: Supplementary file 5 [file wellcomeopenres-2-15254-s0004.tgz › 92ca97e2-8e21-4253-8120-cd31d5220ad6.tif]
