# Supplementary material for: The age of heterozygous telomerase mutant parents influences the adult phenotype of their offspring irrespective of genotype in zebrafish
Source: Wellcome Open Res. 2018 Feb 22;2:77. Originally published 2017 Sep 4. [Version 2] doi: 10.12688/wellcomeopenres.12530.2 (PMC5840683; doi:10.12688/wellcomeopenres.12530.2)
Supplement: Peer review history

# Peer review: Referee response for version 2

**DOI:** 10.21956/wellcomeopenres.15254.r31125

**Published:** 2018-03-06

**Authors:** Noriyoshi Sakai

**Affiliations:** 1 Genetic Strains Research Center, National Institute of Genetics, Mishima, Japan

I agree with the authors' comments that they did not find a statistically significant difference between heterozygous and wild-type fish in fertilization rates and clutch size. It may be difficult to find differences in gonad histology between heterozygous and wild-type in this condition.I think authors properly responded to my comments.

I have read this submission. I believe that I have an appropriate level of expertise to confirm that it is of an acceptable scientific standard.

# Peer review: Referee response for version 2

**DOI:** 10.21956/wellcomeopenres.15254.r31126

**Published:** 2018-03-05

**Authors:** Karl-Lenhard Rudolph

**Affiliations:** 1 Leibniz Institute For Age Research, Jena, Germany

The authors addressed my comments, I am in favor of indexing.

I have read this submission. I believe that I have an appropriate level of expertise to confirm that it is of an acceptable scientific standard.

# Peer review: Referee response for version 1

**DOI:** 10.21956/wellcomeopenres.13568.r26070

**Published:** 2017-10-20

**Authors:** Karl-Lenhard Rudolph

**Affiliations:** 1 Leibniz Institute For Age Research, Jena, Germany

Scahill et al. investigate the influence of the parental age of heterozygous telomerase mutation (Tert ^+/-^) carriers on the phenotype of the offsprings in zebrafish. Tert ^+/-^ zebrafish exhibit a shortened lifespan, reduced body mass, male infertility, and sex bias towards males in the offsprings. These genotypes were aggravated by the parental age of Tert ^+/-^ fish but did not correlate with shorter telomeres. The authors conclude that Tert mutation heterozygosity influences phenotypes in the next generation in a telomere length independent manner.

 This is a very interesting and well-designed study. I have a few points of criticism that should be addressed:

1. The study investigates the phenotype of the offspring in one tree of families and the telomere length in another tree following the same mating scheme from the grandparent generation. The telomere length is measured by sequencing – a new method. The Box plot analysis of mean telomere length shows high spreads. Is it possible that by chance difference in telomere length appear in the offspring generation by selection of germcells with longer or shorter telomeres in the grandparent and parent generation? Can the authors provide some control that the sequencing analysis of telomere DNA content correlates well with telomere length analysis by classical methods such as Southern blot on fin clips?
2. The authors conclude that parental age of Tert mutation carriers influence phenotypes in offspring in a telomere length independent manner. Parental age effects mtations have been described in humans. Is it possible that short telomeres select for genetically or epigenetically altered germline stem cell clones that influence phenotypes in the offspring generation. This selection process may also select for genetically/epigenetically altered germline stem cells with longer telomeres. Even if the telomeres in the offspring would not show a correlation of telomere shortening with the occurrence of phenotypes, it would still be a telomere shortening induced phenotype as the process of germline stem cell selection would triggered by short telomere in the parental germline in an age dependent manner.
3. The authors discuss that the “accumulation of DNA damage could however be independent of telomere length as it has been shown telomere associated DNA-damage response proteins do not differ in length from those that are negative for DNA damage markers (51).” This paper investigates the chromatin response (de-compaction) to telomere uncapping induced by Trf1 and Trf2 deletion. This paper does not investigate the formation of DNA damage foci in response to telomere shortening – the most relevant process in call aging. There is instead good evidence that the induction of DNA damage responses is indeed telomere shortening dependent and occurs at the critically short telomeres, as the authors discuss in the following paragraph of the discussion. The cited part of the discussion should be corrected as it is misleading.

I have read this submission. I believe that I have an appropriate level of expertise to confirm that it is of an acceptable scientific standard, however I have significant reservations, as outlined above.

# Response

**Published:** 2018-02-12

**Authors:** Elisabeth Busch-Nentwich

**Thank you for reviewing our manuscript and your insightful comments. Please find our point-by-point response below.**

Scahill et al. investigate the influence of the parental age of heterozygous telomerase mutation (Tert ^+/-^) carriers on the phenotype of the offsprings in zebrafish. Tert ^+/-^ zebrafish exhibit a shortened lifespan, reduced body mass, male infertility, and sex bias towards males in the offsprings. These genotypes were aggravated by the parental age of Tert ^+/-^ fish but did not correlate with shorter telomeres. The authors conclude that Tert mutation heterozygosity influences phenotypes in the next generation in a telomere length independent manner.

This is a very interesting and well-designed study.

**Response: Thank you for this positive assessment of our work.**

I have a few points of criticism that should be addressed:

1. The study investigates the phenotype of the offspring in one tree of families and the telomere length in another tree following the same mating scheme from the grandparent generation.

**Response: The telomere length measurements in Figure 3 were conducted on two families (MR3309 and MR3310) that were also investigated for their sex ratios in Figure 2E.**

The telomere length is measured by sequencing – a new method. The Box plot analysis of mean telomere length shows high spreads. Is it possible that by chance difference in telomere length appear in the offspring generation by selection of germcells with longer or shorter telomeres in the grandparent and parent generation? Can the authors provide some control that the sequencing analysis of telomere DNA content correlates well with telomere length analysis by classical methods such as Southern blot on fin clips?

**Response: Telomere length measurement by Whole Genome Sequencing (WGS) is an established method and has been benchmarked against non-sequencing based methods such as southern blot (** **https://doi.org/10.1093/nar/gku181** **) and qPCR (** **https://doi.org/10.1371/journal.pone.0125201** **) previously. We apologise for not making this more explicit before, and have added a sentence to introduce the method with references in the results section. The spread of telomere lengths is the same (at 4-6 kb) for MR2884 +/- parents as that found in human samples (** **https://doi.org/10.1093/nar/gku181** **) and smaller for the rest. However, we absolutely agree that it is perfectly possible (and in fact likely) that the overall mean telomere length of the investigated families is determined through sampling from a distribution by descent from two parents. This is supported by our finding that telomere length in the wild-type and heterozygous offspring lies in the range of their parental generation. Therefore our assertions still stand, that 1) absolute mean telomere length is independent from the age of the parents and 2) mean telomere length cannot explain the morphological phenotypes in offspring from old parents. We have added the following sentence to the discussion to acknowledge the role of sampling from a distribution of telomere lengths: “More specifically, it is possible that from the distribution of mean telomere lengths in the grandparents, individuals with longer telomeres were crossed to produce MR3310 and an individual with by chance shorter telomeres was crossed to produce MR2884. This notion is supported by the finding that telomere length in the wild-type and heterozygous offspring lies in the range of their respective parents, but is different between the families despite being of the same age.”**

****

2. The authors conclude that parental age of Tert mutation carriers influence phenotypes in offspring in a telomere length independent manner. Parental age effects mtations have been described in humans. Is it possible that short telomeres select for genetically or epigenetically altered germline stem cell clones that influence phenotypes in the offspring generation. This selection process may also select for genetically/epigenetically altered germline stem cells with longer telomeres. Even if the telomeres in the offspring would not show a correlation of telomere shortening with the occurrence of phenotypes, it would still be a telomere shortening induced phenotype as the process of germline stem cell selection would triggered by short telomere in the parental germline in an age dependent manner.

**Response: This is a very interesting hypothesis, however currently we do not have evidence to support it. Although it is possible that short telomeres in the parental germline may select for genetically or epigenetically altered germline stem cell clones, our results do not indicate that older parents have shorter telomeres. However we only measured telomere lengths in the caudal fin, and it remains possible that telomere lengths in the adult germline are shorter in older *tert *heterozygous fish. Investigating this hypothesis will require telomere length assessment of gonads in a future study.**

The authors discuss that the “accumulation of DNA damage could however be independent of telomere length as it has been shown telomere associated DNA-damage response proteins do not differ in length from those that are negative for DNA damage markers (51).” This paper investigates the chromatin response (de-compaction) to telomere uncapping induced by Trf1 and Trf2 deletion. This paper does not investigate the formation of DNA damage foci in response to telomere shortening – the most relevant process in call aging. There is instead good evidence that the induction of DNA damage responses is indeed telomere shortening dependent and occurs at the critically short telomeres, as the authors discuss in the following paragraph of the discussion. The cited part of the discussion should be corrected as it is misleading.

**Response: We apologise for this error and have removed this sentence from the discussion.**

# Peer review: Referee response for version 1

**DOI:** 10.21956/wellcomeopenres.13568.r26482

**Published:** 2017-10-16

**Authors:** Noriyoshi Sakai

**Affiliations:** 1 Genetic Strains Research Center, National Institute of Genetics, Mishima, Japan

Scahill *et al.* report unique phenotypes of *tert* mutant zebrafish. The mutant homozygotes die prematurely and the males become prematurely infertile. Interestingly, the age of heterozygous parents influences the adult phenotype of their offspring. The authors estimated average telomere lengths by whole genome sequencing data, and conclude that the phenotypes of offspring from older parents cannot be explained by telomere length. These are findings of importance to researchers in the field.

 I have a concern about condition of fertilized eggs from heterozygous mutant parents. During maintenance of zebrafish lines we experience that old parents, especially in the female, produce non-healthy eggs, some of which develop to small the adults. The clutch often shows the male sex bias. Is there any possibility that the adult phenotypes of offspring from heterozygous mutants are from defects in oocytes of aged heterozygous mutants because the heterozygous mutants seem to age faster than the wild-type? Alternatively, germ cells of the heterozygous may spoil faster than those of the wild-type. I recommend to add histology of gonads in heterozygous mutants and data about the numbers of spawning eggs, fertilized eggs, and developed embryos to ~3 dpf in each intercross.

 In addition, authors should describe how offspring is raised. The homozygous mutants display phenotypes of a reduction in head and a short tail. The phenotypes may lead to less eating compared to the wild-type and the heterozygotes when they are in the same dish. This condition may cause the male sex bias of the homozygous mutants.

I have read this submission. I believe that I have an appropriate level of expertise to confirm that it is of an acceptable scientific standard, however I have significant reservations, as outlined above.

# Response

**Published:** 2018-02-12

**Authors:** Elisabeth Busch-Nentwich

**We are grateful for the effort taken to review our manuscript and the helpful comments and questions. Please find our point-by-point response below.**

 Scahill *et al.* report unique phenotypes of *tert* mutant zebrafish. The mutant homozygotes die prematurely and the males become prematurely infertile. Interestingly, the age of heterozygous parents influences the adult phenotype of their offspring. The authors estimated average telomere lengths by whole genome sequencing data, and conclude that the phenotypes of offspring from older parents cannot be explained by telomere length. These are findings of importance to researchers in the field.

**Response: We would like to thank you for concluding that this work is of importance to the field.**

 I have a concern about condition of fertilized eggs from heterozygous mutant parents. During maintenance of zebrafish lines we experience that old parents, especially in the female, produce non-healthy eggs, some of which develop to small the adults. The clutch often shows the male sex bias. Is there any possibility that the adult phenotypes of offspring from heterozygous mutants are from defects in oocytes of aged heterozygous mutants because the heterozygous mutants seem to age faster than the wild-type? Alternatively, germ cells of the heterozygous may spoil faster than those of the wild-type. I recommend to add histology of gonads in heterozygous mutants and data about the numbers of spawning eggs, fertilized eggs, and developed embryos to ~3 dpf in each intercross.

**Response: The reviewer is concerned that the heterozygous fish might be aging prematurely and produce unhealthy oocytes that affect the adult phenotype. We do not find any overt signs of premature aging of the heterozygous fish such as reduced survival rates (Figure 2D and Supplementary Figure S3) or morphological signs of premature aging. However, following the suggestion of the reviewer, we have assessed the egg production and quality of heterozygous females over three months in comparison to wild-type siblings. We do not find a statistically significant difference between heterozygous and wild-type fish in fertilisation rates and clutch size and no deterioration over time (Supplementary Figure 4A). Heterozygous and wild-type females are both capable of laying in excess of 900 eggs per mating. Phenotypic analysis of multiple clutches derived from heterozygous and wild-type intercrosses reveals no difference (Welch two sample t-test, p-val=0.49) in the number of abnormal embryos up to 5 dpf. This is included in the results section: “Intercrosses from heterozygous fish (965 embryos from 3 separate clutches) showed no difference in the number of phenotypic embryos compared to wild-type sibling intercrosses (614 embryos from 4 separate clutches).” This is in contrast to a mutant we have recently published where egg quality diminishes over time (Scahill et al., 2017, DOI: 10.1371/journal.pgen.1006959).**

**Gonad histology might identify differences that are not reflected in egg production and quality. For a meaningful result this would require sampling gonads from young wild-type females and then again their aged siblings to establish a baseline catalogue of normal gonad morphology in young and old wild-type fish. The same process would be required for *tert* heterozygous and homozygous females. We feel that the required time frame and amount of work goes beyond the scope of this publication. Furthermore we are concerned that the lack of an overt oogenesis defect would make it difficult to justify sacrificing such a substantial number of adult fish.**

**Finally, in our facility we haven’t made the same observations as the reviewer that old fish generally give rise to smaller fish and a male skew, although we agree that productivity tends to decrease with increasing age of females. As we show in Figure 2G, in contrast to offspring from 16 month old *tert* heterozygous parents, offspring from very old (30 months) wild-type fish do not show reduced body weight although we do see increased variance. To also systematically assess the impact of parental age on sex ratios, we have used sex ratio data routinely collected in our facility. The analysis of sex ratios across 74 different lines in relation to the age of their parents (Supplementary Figure 4B) finds no correlation of skewed sex ratios with either the paternal or maternal age. We would like to thank the reviewer for raising these questions, since we feel that these additional experiments have strengthened our conclusion that *tert* heterozygosity interacts with age.**

 In addition, authors should describe how offspring is raised. The homozygous mutants display phenotypes of a reduction in head and a short tail. The phenotypes may lead to less eating compared to the wild-type and the heterozygotes when they are in the same dish. This condition may cause the male sex bias of the homozygous mutants.

**Response: The phenotypic embryo in Figure 1F is derived from *tert-/-* intercrosses, not *tert+/-* parents, and is therefore a maternal-zygotic (MZ *tert*) mutant. We have changed the labelling to make this more explicit. Generally, embryos displaying such strong early morphological phenotypes do not survive beyond 3-5 dpf. The clutches raised and analysed for this study were all derived from heterozygous parents. Only morphologically normal larvae with filled swim bladders were raised for this study. We have emphasised this in the methods section.**
